# Gut to lung translocation and antibiotic mediated selection shape the dynamics of *Pseudomonas aeruginosa* in an ICU patient

**DOI:** 10.1038/s41467-022-34101-2

**Published:** 2022-11-22

**Authors:** Rachel M. Wheatley, Julio Diaz Caballero, Thomas E. van der Schalk, Fien H. R. De Winter, Liam P. Shaw, Natalia Kapel, Claudia Recanatini, Leen Timbermont, Jan Kluytmans, Mark Esser, Alicia Lacoma, Cristina Prat-Aymerich, Antonio Oliver, Samir Kumar-Singh, Surbhi Malhotra-Kumar, R. Craig MacLean

**Affiliations:** 1 University of Oxford, Department of Biology, Oxford, UK; 2 Laboratory of Medical Microbiology, Vaccine and Infectious Disease Institute, Faculty of Medicine, University of Antwerp, Wilrijk, Belgium; 3 Molecular Pathology Group, Laboratory of Cell Biology and Histology, Faculty of Medicine, University of Antwerp, Wilrijk, Belgium; 4 Julius Center for Health Sciences and Primary Care, University Medical Center Utrecht, Utrecht University, Utrecht, The Netherlands; 5 Microbial Sciences, BioPharmaceuticals R&D, AstraZeneca, Gaithersburg, MD USA; 6 Microbiology Department, Hospital Universitari Germans Trias i Pujol, Institut d’Investigació Germans Trias i Pujol, CIBER Enfermedades Respiratorias, Universitat Autònoma de Barcelona, Badalona, Spain; 7 Servicio de Microbiología, Hospital Universitari Son Espases, Instituto de Investigación Sanitaria Illes Balears (IdISBa), Palma de Mallorca, Spain

**Keywords:** Antimicrobials, Infection, Evolution, Antimicrobial resistance

## Abstract

Bacteria have the potential to translocate between sites in the human body, but the dynamics and consequences of within-host bacterial migration remain poorly understood. Here we investigate the link between gut and lung *Pseudomonas aeruginosa* populations in an intensively sampled ICU patient using a combination of genomics, isolate phenotyping, host immunity profiling, and clinical data. Crucially, we show that lung colonization in the ICU was driven by the translocation of *P. aeruginosa* from the gut. Meropenem treatment for a suspected urinary tract infection selected for elevated resistance in both the gut and lung. However, resistance was driven by parallel evolution in the gut and lung coupled with organ specific selective pressures, and translocation had only a minor impact on AMR. These findings suggest that reducing intestinal colonization of *Pseudomonas* may be an effective way to prevent lung infections in critically ill patients.

## Abstract (web summary)

In this paper the authors show that the pathogenic bacterium *Pseudomonas aeruginosa* migrates between the gut and lungs of an ICU patient, and that differential evolutionary responses to antibiotic treatment occur in these organs.

## Introduction

Bacteria often colonise multiple anatomical sites in human hosts, but the dynamics of within-host translocation and its consequences for pathogenesis and host adaptation remain poorly understood^1–3^. For example, advances in microbiome profiling methods have shown that the gut microbiome can transmit to the lungs of critically ill patients^4,5^, and translocation is associated with poorer outcomes in mechanically ventilated patients^6^. While gut-to-lung translocation has been demonstrated at the microbiome level, the dynamics and consequences of translocation for individual pathogens and antibiotic resistance are not well understood.

*P. aeruginosa* is an opportunistic pathogen that is a major cause of healthcare-associated infections worldwide^7,8^, most notably in patients with compromised immunity^9,10^. *Pseudomonas* is not considered to be a typical member of the gut microbiome, and intestinal colonisation with *Pseudomonas* is associated with an increased risk of developing lung infections^11–13^ and mortality^14^. Gut colonisation usually precedes lung infection, and the same strain is often found in the gut and lungs, suggesting that the gut acts as a reservoir of *Pseudomonas* that can be transmitted to the lung and other infection sites^15–17^. However, direct evidence for gut-to-lung transmission of *P. aeruginosa* is lacking, and it is possible that intestinal carriage simply reflects an innate susceptibility to *Pseudomonas* infection or proximity to a source of *Pseudomonas* that can independently colonise the lung and gut.

One of the major challenges of dealing with *Pseudomonas aeruginosa* is antibiotic resistance. *P. aeruginosa* has high levels of intrinsic resistance to antibiotics^18–20^ and a remarkable ability to evolve resistance de novo in hospitalised patients^21–23^. In classic population genetic models, migration can accelerate evolutionary adaptation at a local scale by increasing the genetic diversity that selection acts on^24^. In this case, translocation could play a role in antibiotic resistance by moving resistance determinants between bacterial colonisation sites. In an extreme example, bacterial populations in one organ (for example, the gut) could act as a source of resistant mutants that are then disseminated to other organs (i.e. the lung).

To test the importance of gut-to-lung transmission in *Pseudomonas* colonisation and antimicrobial resistance (AMR), we carried out an in-depth case study on a single intensively sampled ICU patient over a 30-day period. We used phylogenetic approaches to test for translocation, and a combination of genomic and phenotypic methods to study the link between AMR and within-host transmission.

## Results

### Clinical timeline

**Fig. 1:**
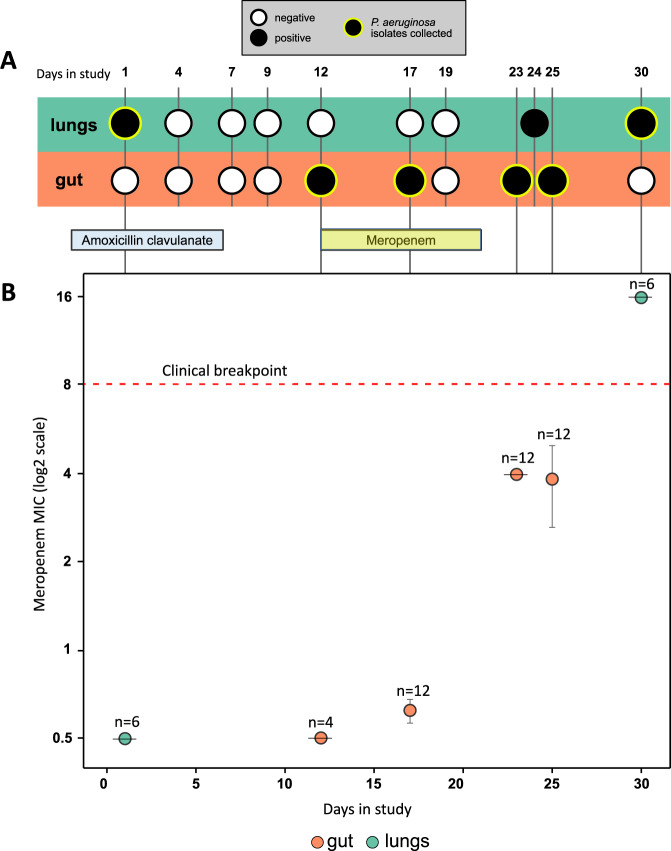
Clinical timeline and resistance phenotyping. **A** Timeline of patient sampling, showing samples that tested positive or negative for *P. aeruginosa* colonisation by culturing. Sampling points from which isolates were collected are highlighted with a green ring. The patient was treated with amoxicillin clavulanate from 2 days prior to enrolment until day 6 and with meropenem from day 12 to day 21. **B** Meropenem minimum inhibitory concentration (MIC) (mean ± s.e.m μg/mL) for isolates (*n* = 4–12, as labelled on plot) from each sampling point from the gut (orange) and lung (green). Each isolate had a median meropenem MIC calculated from *n* = 3 biologically independent replicates. Meropenem resistance increased over time, and *P. aeruginosa* isolates from the final lung sample were above the EUCAST clinical breakpoint for meropenem resistance (red dashed line). Amoxicillin clavulanate resistance was not measured as this antibiotic is not active against *P. aeruginosa*. Source data are provided as a Source Data file.

The focal patient was admitted to ICU of Hospital Universitari Germans Trias i Pujol in Badalona, Spain with a primary diagnosis of seizure. Mechanical ventilation was started on ICU admission and was continued for a total duration of 39 days. The patient was immediately treated with amoxicillin clavulanate, which is not active against *P. aeruginosa*, due to suspected aspiration of oropharyngeal or gastric contents into the lower respiratory tract (bronchoaspiration). The patient was enrolled in ASPIRE-ICU trial^25^ at 48 h post admission (hereafter day 1). Meropenem was started on day 12 and continued for 10 days to treat a suspected urinary tract infection. Over the course of stay in ICU, a total of 52 *P. aeruginosa* isolates were collected, from endotracheal aspirate (ETA) samples (*n* = 12) and peri-anal swabs (*n* = 40) up until day 30 (Fig. 1), which was the end-point of the ASPIRE-ICU trial. Culture screening of patient blood samples from day 2, day 11 and day 21 all reported no growth of *P. aeruginosa*. *P. aeruginosa* colonisation was detected in the lungs at day 1. Gut colonisation was detected following meropenem treatment, and meropenem resistant *P. aeruginosa* ultimately colonised the lung. This complex clinical timeline suggests that translocation between the gut and lung may have occurred (Fig. 1), but clinical data and isolate phenotypes alone provide limited insights into the underlying drivers of within-host translocation and AMR.

### Genomic insights into pathogen colonisation and evolution

To characterise the genetic diversity within this patient, we used long and short read sequencing to construct a hybrid assembly for a single isolate, yielding a ~6.3 Mb ST782 reference genome distributed across 5 contigs. Short-read sequences of lung (*n* = 12) and gut (*n* = 40) isolates were mapped to this reference genome, and we identified polymorphic SNPs (*n* = 17), indels (*n* = 7), and variation in presence/absence of a 190 kb genomic island (Supplementary Table 1 and Supplementary Fig. 1).

**Fig. 2:**
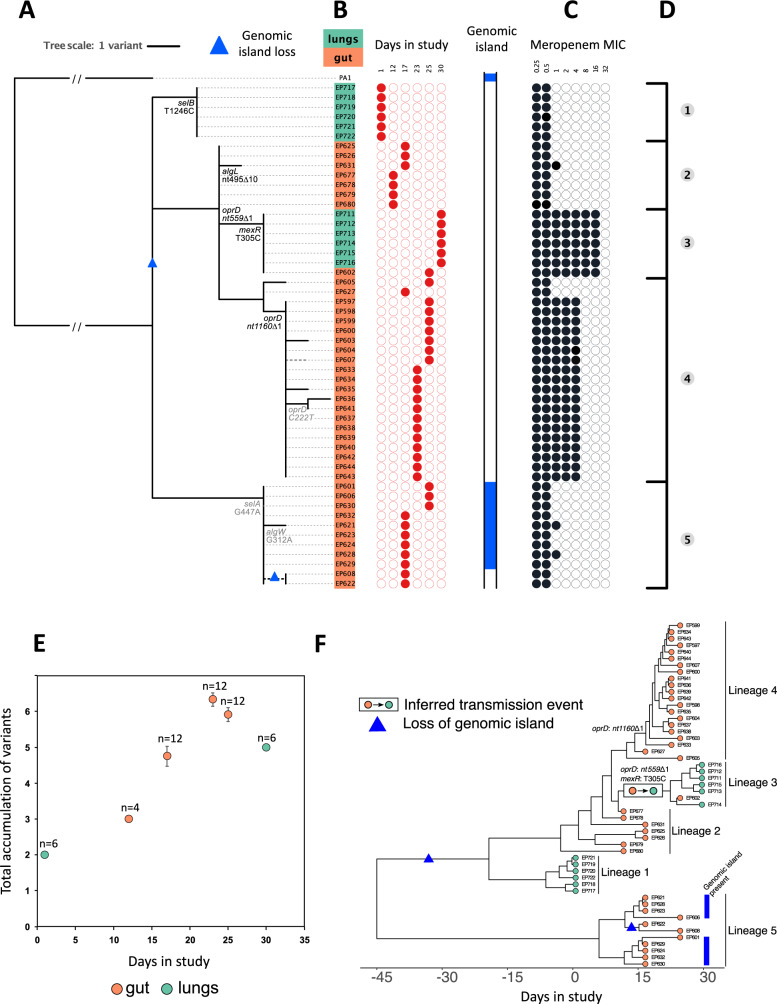
Genome sequencing and phylogenetic analysis. **A** Phylogenetic reconstruction of lung (*n* = 12) and gut (*n* = 40) isolates rooted using *P. aeruginosa* PA1, another ST782 clinical isolate sampled from a respiratory tract infection^72^, as the outgroup. Putatively adaptive polymorphisms in genes or pathways showing parallel evolution are annotated on the phylogeny. Protein altering mutations are shown in black and silent mutations are shown in light grey. A polymorphism in a known multi-drug efflux pump regulator (*mexR*) is also highlighted. Variation in the presence/absence of a 190kB genomic island is shown, and inferred losses of the genomic island are identified with blue triangles in the tree. **B** Isolate name, lung (green) or gut (orange) origin, and day in study of collection. **C** Susceptibility to meropenem for each isolate is presented with filled black circles in log_2_ scale of the minimum inhibitory concentration (MIC). **D** The topology of the tree suggested five distinct groups based on the identification of small polymorphisms. **E** The accumulation of variants over time (mean ± s.e for isolates (*n* = 4–12, as labelled on plot) from each sampling point from the gut (orange) and lung (green) suggests within-host evolution of a clone rather than recurrent episodes of colonisation. **F** Dated genealogy of isolates constructed with BactDating^73^. The inferred instance of gut to lung transmission and the acquisition of meropenem resistance mutations have been annotated on the genealogy. Source data are provided as a Source Data file.

The genetic diversity found in this patient could reflect either (i) recurrent colonisation/infection by multiple clones or (ii) within-host evolution of a single clone. To discriminate between these processes, we reconstructed the phylogeny of isolates using *P. aeruginosa* PA1, a closely related ST782 genome, as an outgroup (Fig. 2A–D). All of the isolates were closely related and the number of variants per isolate correlated strongly with the day of isolate collection, supporting the idea that within-patient diversity emerged as a result of in situ evolution of a single founding clone (Fig. 2E; *r*^2^ = 0.62, *F*_1,50_ = 82, *P* < 0.0001). No other patients within the ASPIRE-ICU cohort at this hospital were colonised by *P. aeruginosa* ST782 during the trial, providing further support for the within-host diversification as opposed to repeated colonisation.

We estimated the onset of colonisation using a time-scaled genealogy of isolates with the most-recent common ancestor (MRCA) of all isolates predicted to be at least 3 weeks prior to ICU admission (Fig. 2F; Supplementary Fig. 2; MRCA from BactDating: 22–74 days before day 0)^26^.The rate of evolution in this patient was ~18 ± 10 SNPs/year^26^, which is higher than the typical evolutionary rate of bacterial pathogens of 1–10 SNPs/year^3^. However, this elevated evolutionary rate is in line with the rate reported from another patient in this trial^23^, highlighting the high in vivo mutation rate of *P. aeruginosa* in critically ill patients. This phylogenetic approach, which strongly supports continuous host colonisation, suggests that culture-based approaches (i.e. Fig. 1A) have limited ability to detect *P.aeruginosa* colonisation.

Signatures of parallel evolution provide a simple way to identify putative beneficial mutations that underpin adaptation to the novel environment of the human host^3,27^. Parallel evolution occurred in 3 genes or operons that have functional roles in resistance to carbapenem antibiotics (*oprD*)^27^, alginate biosynthesis (*algW, algL*)^28^, and selenocysteine biosynthesis (*selA and selB*)^29^. These putative pathoadaptive mutations accounted for 7 of the 24 variants, providing strong evidence for rapid adaptation to the host environment. Interestingly, 3 of these 7 mutations were synonymous, suggesting that transcription efficiency may have been a key target of selection^30^. The presence of the variable genomic island in the outgroup and in lung isolates (Fig. 2, lineage 5) implies that island was lost on two independent occasions, suggesting that loss of this element was adaptive. Inferring the selective advantage of large scale deletions is difficult, but it is worth noting that this island carries pyoverdine biosynthesis genes that are selected against in the host environment^31^. Isolates in possession of the genomic island showed similar levels of meropenem resistance (~0.5 μg/mL MIC) to isolates from the same phylogenetic lineage (lineage 5) with loss of the genomic island (Fig. 2), suggesting that the loss of this island was not driven by antibiotic treatment.

Bacterial phylogenies are a powerful tool to detect transmission events, particularly when combined with isolate sampling dates^3,32,33^. To reconstruct within-host translocation, we used our time scaled isolate genealogy to infer translocation events (Fig. 2F). Secondary lung colonisation was driven by the growth of a clone with mutations in the *oprD* porin, which is a key carbapenem sensitivity determinant, and *mexR*, which regulates the expression the MexAB-OprM multi-drug efflux pump^34^ (Fig. 2A – lineage 3). This lineage is nested within a broader clade of gut isolates, providing strong evidence of gut to lung transmission (Fig. 2F). The dated genealogy suggests the MRCA of lineage 3 existed between day 18 and day 24 (Supplementary Fig. 2)^26^, giving an approximate time frame for gut to lung translocation. However, this genealogy does not provide any insight into whether lineage 3 acquired the carbapenem resistance mutations before or after transmission to the lung. Interestingly, a single lineage 3 isolate was recovered from the gut. The presence of this lineage in the lung and gut implies that either meropenem resistance evolved in the gut prior to transmission to the lung, or that this lineage secondarily transmitted from the lung to the gut after evolving meropenem resistance in the lung.

Beyond this clear-cut case of gut to lung translocation, inferred patterns of translocation depend strongly on the assumptions made regarding initial colonisation by the ancestral clone. If *Pseudomonas* initially colonised the gut, then the phylogeny implies that initial lung colonisation by lineage 1 was driven by an earlier gut-to-lung translocation event that could have been associated with broncoaspiration upon ICU admission. Alternatively, the ancestral clone may have colonised the lungs. Under this model of host colonisation, the phylogeny suggests that lung to gut transmission occurred in lineages 2 and 5, implying that at least one episode of translocation occurred at some point prior to ICU admission. Finally, it is possible that the ancestral clone independently colonised both the lungs and gut. According to this model of host colonisation, it is not necessary to invoke any additional translocation (beyond the clear gut to lung translocation in lineage 3) in order to explain the phylogenetic distribution of lung and gut isolates. Unfortunately, it is not possible to clearly differentiate between these models because the lack of pre-ICU admission isolates hinders our ability to reconstruct the early history of *Pseudomonas* colonisation in this patient. All three models of host colonisation lead to a similar number of inferred translocation events (1–3), suggesting that they are similarly parsimonious from a phylogenetic perspective. Culturing patient samples found evidence of lung colonisation prior to gut colonisation (Fig. 1A), but we argue that this culture data provides limited insights into early host colonisation, which likely occurred >20 days prior to ICU admission. A further challenge of using culture data to infer host colonisation is that the limits of detection from culturing samples from different tissues are unknown, and it is conceivable that ETA samples and peri-anal swabs simply differ in their sensitivity to detect *Pseudomonas* in the lung and gut.

### Immune response to lung colonisation

**Fig. 3:**
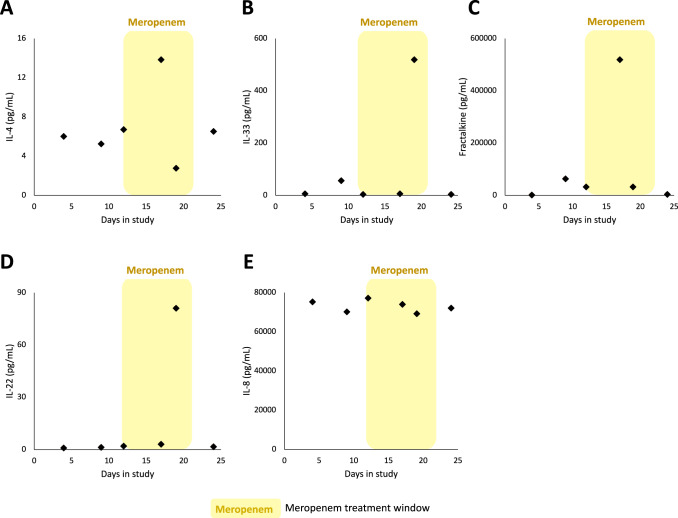
Cytokine concentrations were measured in ETA samples collected over the course of the study at days 4, 9, 12, 17, 19 and 24. The following cytokines were measured: **A** IL-4, **B** IL-33, **C** Fractalkine, **D** IL-22, **E** IL-8. Yellow shading indicates meropenem treatment window on timeline (day 12 to day 21). Source data are provided as a Source Data file.

Lung colonisation provides *P. aeruginosa* with the opportunity to establish infection by adhering to the mucosal surface and penetrating the epithelial barrier, leading to the development of pneumonia, which is associated with a very high mortality rate in ICU patients^7^. To investigate the role of host immunity in preventing infection, we measured the abundance of a panel of host immune effectors in samples of endotracheal aspirate (ETA; Fig. 3). The dated genealogy suggests that secondary lung colonisation by lineage 3 occurred between day 18 and day 24. Interestingly, ETA samples from days 17 and 19 were associated with spikes in the expression of IL-33, Fractalkine, and IL-4^35,36^, which have previously been to shown to enhance the clearance of *P. aeruginosa*^37^. Colonisation was also associated with a > 10-fold increase in the concentration of IL-22 (day 19 sample), which protects against infections caused by attaching and effacing bacterial pathogens by increasing mucous production and by limiting excessive inflammation mediated by neutrophil influx^38,39^. Measuring cytokine levels in ETA samples from the lungs provides a direct measurement of immune response, but one concern over this approach is that it is possible for individual samples to give high concentrations of all cytokines, for example as a result of patient dehydration. However, in this case levels of IL-8 remained essentially constant across samples, supporting the idea that spikes of protective cytokines were not an artefact (Fig. 3E). These cytokine data provide further support of the idea that secondary lung colonisation occurred at some point between day 12 and day 17, and they suggest that the host immune response may have prevented colonisation from progressing to pneumonia.

### Drivers of antibiotic resistance

**Fig. 4:**
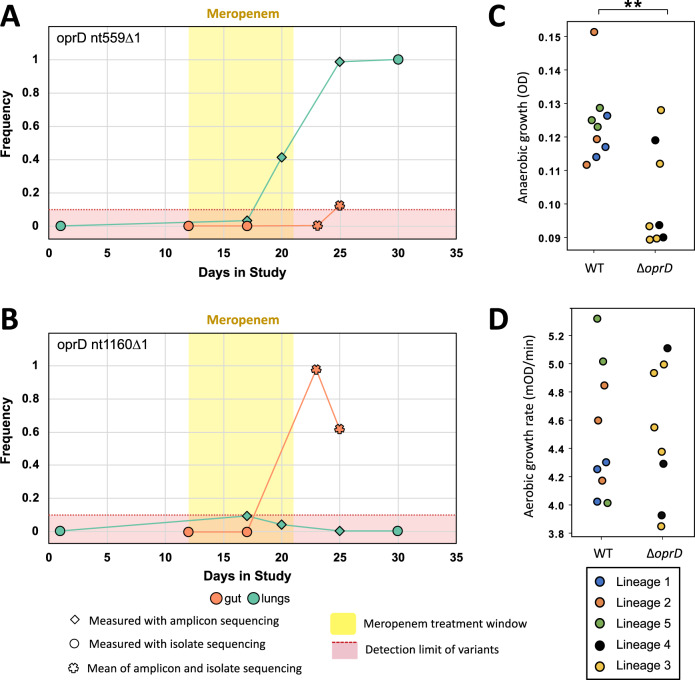
Evolution and transmission of meropenem resistance. **A**, **B** Dynamics of *oprD* variants, as determined by isolate (circle) and *oprD* amplicon (diamond) sequencing data. For the two sampling points (gut day 23 and gut day 25) where both amplicon and isolate sequencing was carried out, the mean of the two frequencies is shown (star). Measurements of SNP frequency from isolate sequencing and amplicon sequencing were strongly correlated (*R*^2^ = 0.9963; Supplementary Fig. 3). The yellow area shows the window of meropenem treatment and the red area shows a conservative minimum detection limit of variants from amplicon sequencing due to the error rate of nanopore sequencing. **C**, **D** Growth of isolates with an o*prD* variant (Δ o*prD*) compared to isolates with the wild-type *oprD* background. Anaerobic growth (**C**) was measured as OD_595_ after 72 hours growth in anaerobic broth. Values plotted for each point (i.e. isolate) are calculated from *n* = 3 biologically independent replicates (Source Data), and at least three isolates from each lineage were measured. Aerobic growth (**D**) was measured as exponential growth rate in standard culture conditions. Isolates are colour coded according to phylogenetic lineage, as defined in Fig. 2. Values plotted for each point (i.e. isolate) are calculated from *n* = 7 biologically independent replicates (Source Data), and at least three isolates from each lineage were measured. *oprD* mutations were associated with impaired growth under anaerobic conditions (*P* = 0.010), but not aerobic conditions (*P* = 0.950), as judged by a nested ANOVA. Data from isolates from different lineages are shown together because fitness measures did not differ between lineages nested within *oprD* genotype (*P* > 0.5). All statistical tests for this analysis are two-tailed. Source data are provided as a Source Data file.

*P. aeruginosa* has high levels of intrinsic antibiotic resistance and a remarkable ability to evolve increased resistance under antibiotic treatment^18,21^. Given the possibility of translocation between the gut and lung, we next sought to understand the relative contributions of migration, mutation and selection to the origin and spread of meropenem resistance in this patient. The phylogeny clearly shows that elevated meropenem resistance evolved on 2 separate occasions due to mutations in *oprD* and *mexR* (Fig. 2, lineages 3 and 4). However, it is challenging to follow the dynamics of meropenem resistance mutations using isolates alone due to the limited number of isolates sequenced (*n* = 52) and the gaps in the sampling of isolates. To try and overcome this problem, we combined isolate sequencing data with amplicon sequencing of *oprD* using DNA extracted directly from ETA samples and peri-anal swabs, some of which were not screened for isolates according to ASPIRE-ICU protocol (Fig. 4A, B). Amplicon sequencing revealed that *Pseudomonas* was present in the lung from day 17 onwards, which coincides with the inferred time of secondary lung colonisation from the isolate phylogeny (Fig. 2F) and cytokine profiling (Fig. 3). *oprD* amplicon sequencing from DNA isolated from peri-anal swabs was less successful for reasons that are not clear, and we only obtained amplicon sequencing data from days 23 and 25. Importantly, the frequency of *oprD* variants measured by amplicon and isolate sequencing from samples that yielded both isolates and *Pseudomonas* DNA was essentially identical (Supplementary Fig. 3), validating the use of amplicon sequencing to measure changes in allele frequency.

The most common *oprD* mutation in sequenced isolates (nt1160∆1) arose in the main clade of intestinal isolates (Fig. 2, lineage 4) and was never convincingly detected in lung samples (Fig. 4B). This combination of isolate and amplicon sequencing results provides strong evidence that this mutation arose in the gut and swept to near fixation under meropenem treatment. The frequency of this mutation ultimately declined in the gut due to the expansion of a carbapenem sensitive lineage (Fig. 2A – lineage 5). We speculate that this lineage may have survived carbapenem treatment by either colonising a region of the gut with low carbapenem toxicity or by forming persister cells.

Isolate sequencing revealed a second frameshift mutation in *oprD* (nt559∆1) that was linked to a mutation in the *mexR* effux pump transcriptional regulator. Isolate sequencing revealed the presence of the *oprD* nt559∆1/*mexR*T305C lineage in gut samples from day 25 and lung samples from day 30, providing strong evidence that this resistant lineage transmitted between colonisation sites. Amplicon sequencing first detected this mutation as a polymorphism (frequency approx. 20%) in the lung at day 20 (Fig. 4A), suggesting that this mutation arose in the lung following the transmission of lineage 3 from the gut. According to this model, the presence of rare *oprD* nt559∆1 variants (in isolates and amplicons) at day 25 reflects secondary lung to gut transmission. However, we emphasise that it is not possible to completely exclude the possibility that *oprD* nt559∆1 arose in the gut and then transmitted to the lung where it spread rapidly by positive selection.

Antibiotic concentrations vary between host tissues, and it is unclear to what extent pathogen populations adapt to local variation in selective pressures associated with antibiotic treatment. Meropenem achieves higher concentrations in lung tissues than in the gut^40^, suggesting that selection for resistance is stronger in the lung than in the gut. Isolates from the lung-associated *oprD* nt559∆1/*mexR*T305C lineage had higher meropenem resistance (MIC = 16 μg/mL, s.e. = 0 μg/mL, *n* = 7) than isolates from the gut-associated *oprD* nt1160∆1 lineage (MIC = 4  μg/mL, s.e. = 0 μg/mL, *n* = 19; Fig. 2C), which is consistent with the idea that selection for meropenem resistance varies between organs.

A key challenge in evolutionary studies of AMR is to understand how resistance can be maintained in pathogen populations in the absence of continued antibiotic use^41,42^. In this case, *oprD* nt559∆1 resistance remained stable in the lung following antibiotic treatment, but the frequency of *oprD* nt1160∆1 declined in the gut. To test the role of selection in the stability of resistance, we measured the growth rate of isolates of all 5 major phylogenetic lineages in anaerobic culture medium (Fig. 4C) and aerobic culture medium (Fig. 4D), which recapitulates one of the physiological differences between the gut and lung. Meropenem resistant lineages (Δ o*prD*) were not associated with decreased growth rate under aerobic conditions, suggesting that the *oprD* and *mexR* mutations have little, if any, associated costs under these conditions (Fig. 4D; *F*_1,12_ = 0.0032, *P* = 0.956). In contrast, both meropenem lineages were associated with decreased growth under anaerobic conditions, suggesting that fitness costs associated with *oprD* mutations drove the loss of resistance in the gut (Fig. 4C; *F*_1,12_ = 9.27, *P* = 0.010).

## Discussion

The goal of this project was to understand the link between gut and lung *Pseudomonas* colonisation in a single patient. By combining clinical and genomic data, we were able to demonstrate a clear cut case of gut to lung transmission while the patient was in ICU. Whilst it is difficult to generalise the findings of a single case study, these findings support the idea that gut to lung transmission may be a major driver of *P. aeruginosa* respiratory tract colonisation in critically ill patients^11,12,43^.

Carbapenem antibiotics such as meropenem are key to the treatment of *P. aeruginosa* infections^23,44,45^, and carbapenem-resistant *P. aeruginosa* has been identified as an important threat by the World Health Organisation and the Centers for Disease Control and Prevention. In this patient, meropenem treatment for a suspected urinary tract infection drove the repeated evolution of resistance, providing a poignant example of the importance of ‘bystander selection’ for AMR^46^. Ultimately, selection led to the emergence of a population of highly resistant bacteria that persisted in the lung in the absence of antibiotic treatment, suggesting that the respiratory tract may act as a source of carbapenem resistant *Pseudomonas* that can transmit to other body sites and potentially to other patients. Isolate sequencing, amplicon sequencing and immunological profiling all support the idea that gut to lung translocation coincided with meropenem treatment. This association may have arisen due to chance, but it is also possible that antibiotic treatment facilitated gut to lung transmission, for example by eliminating commensal lung bacteria that protected against *Pseudomonas* colonisation.

Migration increases genetic variation^24^, suggesting that within-host translocation may accelerate bacterial adaptation to antibiotics^1–3,47–50^. In this case, resistance was driven by the spread of independent lineages in the gut and lung that were adapted to local differences in antibiotic concentration. We found some evidence of translocation of resistant lineages, but the impact of within-host migration on resistance was weak compared to selection, leading to the emergence of a highly structured meropenem resistant pathogen population^1–3,49^. We speculate that the high in vivo mutation rate of *Pseudomonas* was key to shaping local adaptation to antibiotic selection across tissues, and that within-host transmission is likely to provide a more important source of resistance at smaller spatial scales^2^, or when mutation rate is low.

Hospital acquired infections caused by epidemically successful MDR and XDR strains of *P. aeruginosa* have become a serious problem worldwide^19^, and there is an urgent need to develop new antibiotics to treat infections caused by these strains. At the same time, the incredible ability of *Pseudomonas* to evolve resistance to antibiotic treatment^18,21,23,47^ highlights the need to develop novel approaches to prevent or treat *Pseudomonas* infections. Our study suggests that preventing gut colonisation or gut to lung transmission may be an effective strategy for preventing *Pseudomonas* infection in critically ill patients^51–54^.

## Methods

### Clinical timeline

The patient was admitted to the intensive care unit (ICU) of Hospital Universitari Germans Trias i Pujol in Badalona, Spain with a primary diagnosis of seizure. This patient was recruited as part of an observational multicenter European epidemiological cohort study (ASPIRE-ICU^25^), which was conducted according to the principles of the Declaration of Helsinki, in accordance with the Medical Research Involving Human Subjects Act and local guidelines in the participating countries. The study protocol was approved by the Research Ethics Committee of the Germans Trias i Pujol University Hospital and participants gave written informed consent.

At the time of ICU admission, the patient did not suffer from pneumonia or any other active *P. aeruginosa* infection (APACHE-II score = 22 and Glasgow Coma scale = 3). No antibiotic use was reported in the two weeks prior to hospital admission. After 48 h of ICU admission, informed consent was obtained and the patient was enrolled in the ASPIRE-ICU study (day 1)^25^. Mechanical ventilation was started on ICU admission and was continued for a total duration of 39 days. Amoxicillin/clavulanic acid (1000 mg IV q8h for 8 days) was started on ICU admission for bronchoaspiration, the suspected inhalation of oropharyngeal or gastric contents into the lower respiratory tract. Meropenem (1000 mg IV q8h for 10 days) was started on day 12 to treat a suspected urinary tract infection. Patient endotracheal aspirate (ETA) and peri-anal swab samples were collected and screened for *P. aeruginosa* isolates via selective plating until day 30^23^. Patient ETA samples were first blended (30,000 rpm, probe size 8 mm, steps of 10 s, max 60 s in total), diluted 1:1 v/v with Lysomucil (10% Acetylcysteine solution) (Zambon S.A, Belgium) and incubated for 30 min at 37 °C with 10 s vortexing every 15 min. Selective plating to screen for *P. aeruginosa* was carried out using CHROMID *P. aeruginosa* Agar (BioMérieux, France) and blood agar (BBL®Columbia II Agar Base (BD Diagnostics, USA) supplemented with 5% defibrinated horse blood (TCS Bioscience, UK)). Matrix-Assisted Laser Desorption Ionization-Time of Flight Mass Spectrometry (MALDI-TOF MS) was used to identify up to 12 *P. aeruginosa* colonies per sample, which were stored at −80 °C until further use. This resulted in a total of 52 P. aeruginosa isolates collected from endotracheal aspirate samples (*n* = 12) and peri-anal swabs (*n* = 40). In addition, patient blood cultures from day 2, day 11 and day 21 were screened at the local laboratory, all reported no growth (negative for *P. aeruginosa*). The patient was discharged from the ICU and transferred to a general medical ward on day 41.

### Resistance phenotyping

All isolates were grown from glycerol stocks on Luria-Bertani (LB) Miller Agar plates overnight at 37 °C. Single colonies were then inoculated into LB Miller broth for 18–20 h overnight growth at 37 °C with shaking at 225 rpm. Overnight suspensions were serial diluted to ~5 × 10^5^ CFU/mL. Resistance phenotyping to meropenem was carried out as minimum inhibitory concentration (MIC) testing via broth microdilution as defined by EUCAST recommendations^55,56^, with the alteration of LB Miller broth for growth media and the use of *P. aeruginosa* PAO1 as a reference strain. Resistance to meropenem was assayed along a 2-fold dilution series between 0.25–64 μg/mL. We defined growth inhibition as OD_595_  <  0.200 and we calculated the MIC of each isolate as the median MIC score from three biologically independent assays of each isolate (Source Data).

### Growth assays

*P. aeruginosa* isolates were grown from glycerol stocks on LB Miller Agar plates overnight at 37 °C. Single colonies were then inoculated into LB Miller broth for 18–20 h overnight growth at 37 °C with shaking at 225 rpm. Overnight suspensions were serially diluted to an OD_595_ of ~0.05 within the inner 60 wells of a 96-well plate equipped with a lid. To assess growth rate under standard aerobic conditions, isolates were then grown in LB Miller broth at 37 °C and optical density (OD595 nm) measurements were taken at 10-min intervals in a BioTek Synergy 2 microplate reader set to moderate continuous shaking. Growth rate (Vmax; mOD/min) was calculated as the maximum slope of OD versus time over an interval of ten consecutive readings, and we visually inspected plots to confirm that this captured log-phase growth rate. We measured the growth rate of all 52 gut and lung isolates with a minimum of seven biological replicates to assess the relationship between meropenem resistance and fitness (Source Data). Throughout growth assays a media control (to control for contamination) and a PAO1 control (to control for replicate plates) were included. We measured anaerobic growth using an anaerobic jar (Thermo Scientific^TM^ Oxoid^TM^ AnaeroJar^TM^ base jar) system with anaerobic gas generating sachets (Thermo Scientific^TM^ Oxoid^TM^ AnaeroGen^TM^ sachets). An Oxoid Resazurin indicator strip was placed in the jar as an indicator to confirm generation of an anaerobic environment. For growth measurements, single colonies were inoculated into LB Miller broth in the wells of a 96-well plate and placed in the anaerobic jar for 72 h, after which plates were removed and OD_595_ was measured. For the comparison of o*prD* variant (Δ o*prD*) isolates to wild-type *oprD* background (WT *oprD*) isolates, growth measurements were taken for a minimum of three isolates (and a minimum of three biological replicates) selected as representatives from each phylogeny group to generate a mean growth measurement for each Δ o*prD* and WT *oprD* group. To test for an association between *oprD* mutations and impaired growth we used a nested ANOVA that included main effects of *oprD* (ie either WT or Δ o*prD*, 1 df) and phylogenetic lineage nested within *oprD* (5 lineages shown in Figs. 2 and 3 df).

### Illumina sequencing

All isolates were sequenced in the MiSeq or NextSeq illumina platforms yielding a sequencing coverage of 21X–142X. Raw reads were quality controlled with the ILLUMINACLIP (2:30:10) and SLIDINGWINDOW (4:15) in trimmomatic v. 0.39^57^. Quality controlled reads were assembled for each isolate with SPAdes v. 3.13.1^58^ with default parameters. These assemblies were further polished using pilon v. 1.23^59^ with minimum number of flank bases of 10, gap margin of 100,000, and kmer size of 47. Resulting contigs were annotated based on the P. aeruginosa strain UCBPP-PA14^60^ in prokka v. 1.14.0^61^. Each isolate was typed using the Pseudomonas aeruginosa multi-locus sequence typing (MLST) scheme from PubMLST (last accessed on 11.06.2021)^62^.

### Long-read sequencing

Two isolates (EP717 (day 1 lungs) and EP623 (day 17 gut)) were sequenced using the Oxford nanopore MinION platform with a FLO-MIN106 flow-cell and SQK-LSK109 sequencing kit. EP717 had sequencing coverage of 141X and EP623 of 233X. Raw reads were basecalled using guppy v. 0.0.0 + 7969d57 and reads were demultiplexed using qcat v. 1.1.0 (https://github.com/nanoporetech/qcat). Resulting sequencing reads were assembled using unicycler v. 0.4.8^63^, which used SAMtools v. 1.9^64^, pilon v. 1.23^59^, and bowtie2 v. 2.3.5.1^65^, in hybrid mode with respective illumina reads. The EP717 assembly had a N50 of 1,797,327 for a total of 6,217,789 bases distributed in 11 contigs. The EP623 assembly had a N50 of 6,133,283 for a total of 6,330,243 bases distributed in 5 contigs.

### Variant calling

To identify mutations and gene gain/loss during the infection, short-length sequencing reads from each isolate were mapped to each of the long-read de novo assemblies with BWA v. 0.7.17^66^ using the BWA-MEM algorithm. Preliminary SNPs were identified with SAMtools and BCFtools v. 1.9. Low-quality SNPs were filtered out using a two-step SNP calling pipeline, which first identified potential SNPs using the following criteria: (1) Variant Phred quality score of 30 or higher, (2) At least 150 bases away from contig edge or indel, and (3) 20 or more sequencing reads covering the potential SNP position. In the second step, each preliminary SNP was reviewed for evidence of support for the reference or the variant base; at least 80% of reads of Phred quality score of 25 or higher were required to support the final call. An ambiguous call was defined as one with not enough support for the reference or the variant, and, in total, only one non-phylogenetically informative SNP position had ambiguous calls. Indels were identified by the overlap between the HaplotypeCaller of GATK v. 4.1.3.0^67^ and breseq v. 0.34.0^68^. The maximum parsimony phylogeny was constructed based on high-confidence SNPs. To construct a dated genealogy of isolates, we dated the internal nodes of this tree using bactdate in BactDating v1.1.0^26^ (updateRoot = TRUE, minbralen=0.1) after first converting multifurcating nodes to binary nodes. Phylogenies were plotted with ggtree v3.0.4^69^.

The variable genome was surveyed using GenAPI v. 1.098^70^ based on the prokka annotation of the short-read de novo assemblies. The presence or absence of genes in the potential variable genome was reviewed by mapping the sequencing reads to the respective genes with BWA v.0.7.17^66^.

### Amplicon Sequencing of oprD

Amplicon sequencing of the *oprD* gene was carried out to quantify the presence of the two key *oprD* variants observed in the isolate sequencing in whole gDNA samples that were available from the lung and gut of this patient. DNA was extracted from the ETA and peri-anal swab samples using a ZymoBIOMICS DNA Miniprep Kit (Zymo Research, CA USA).

On these extracted gDNA samples, a PCR amplification strategy using barcoded primers to amplify the *oprD* gene (1489 bp product length) and add sample specific DNA barcodes was followed^71^. This was performed with PCR in Q5 High-Fidelity Master Mix (New England BioLabs) and using a universal reverse primer and sample specific forwards primers containing 12 nt barcodes listed in Supplementary Table 2^71^. The temperature profile was 98 °C for 30 s, followed by 30 cycles of 98 °C for 10 s, 70 °C for 30 s, 72 °C for 40 s, followed by a final extension of 72 °C for 2 min. The barcoded *oprD* PCR products were pooled and sequenced on an Oxford nanopore MinION platform using a FLO-MIN106 flow-cell and the SQK-LSK109 Ligation Sequencing kit. Amplicon sequencing raw reads were basecalled using guppy v. 0.0.0 + 7969d57. This yielded 163,766 reads with an estimated read length N50 of 2.64 kb. The data was demultiplexed allowing 2/12 sequencing errors in the barcode sequence and a maximum of 1 error in the downstream and upstream 4-mer. To identify the genotype of each read, we searched for the 11-mer sequence including the variant base and 5 bases downstream and upstream from this position. Using this conservative approach, we recovered 32–43% of the reads (Supplementary Table 3).

### Cytokine measurements

After ETA was blended, 0.5 g of the sample was diluted 1:1 with Sputolysin (Merck, Overijse, Belgium), vortexed and incubated at room temperature for 15 min. Samples were then centrifuged for 5 min at 2000×*g* at room temperature. Supernatant was stored at −80 °C until further processing. Levels of interleukin (IL-)4, IL-33, IL-22, IL-8 and fractalkine were measured with the Mesoscale Discovery platform (Rockville, MD, USA) following the manufacturer’s instructions. In brief, the plate was coated with capturing antibodies for 1 h with shaking incubation at room temperature followed by washing off the plate. Samples were loaded and incubated for 1 h, after which the plate was washed and incubated with detection antibodies. A final wash was performed and MSD reading buffer 2x was applied before reading the plate in the QuickPlex SQ 120 (Rockville, MD, USA).

### Reporting summary

Further information on research design is available in the Nature Research Reporting Summary linked to this article.

## Supplementary information

Supplementary Information

Reporting Summary

## Data Availability

All clinical data analyzed for this patient as part of the study are included in this article. Isolates can be obtained from the corresponding author for research use via an MTA subject to permission from the ASPIRE research committee. The source data are provided with this paper and have been deposited in the Oxford Research Archive for Data (“DOI: 10.5287/bodleian:r1ekRa9wE” [10.5287/bodleian:r1ekRa9wE]). All sequencing data generated in this study has been deposited in the NCBI short-read archive (“PRJNA802704”) and all data on isolates can be found at: “SRR17868883”, “SRR17868884”, “SRR17868885”, “SRR17868886”, “SRR17868887”, “SRR17868888”, “SRR17868889”, “SRR17868890”, “SRR17868891, “SRR17868892”, “SRR17868893”, “SRR17868894”, “SRR17868895”, “SRR17868896”, “SRR17868897”, “SRR17868898”, “SRR17868899”, “SRR17868900”, “SRR17868901”, “SRR17868902”, “SRR17868903”, “SRR17868904”, “SRR17868905”, “SRR17868906”, “SRR17868907”, “SRR17868908”, “SRR17868909”, “SRR17868910”, “SRR17868911”, “SRR17868912”, “SRR17868913”, “SRR17868914”, “SRR17868915”, “SRR17868916”, “SRR17868917”, “SRR17868918”, “SRR17868919”, “SRR17868920”, “SRR17868921”, “SRR17868922”, “SRR17868923”, “SRR17868924”, “SRR17868925”, “SRR17868926”, “SRR17868927”, “SRR17868928”, “SRR17868929”, “SRR17868930”, “SRR17868931”, “SRR17868932”, “SRR17868933”, “SRR17868934” Source data are provided with this paper.

## Source data

Source Data

## Code availability

Code used for analyses are publicly available via Github [“https://github.com/juliofdiaz/Wheatley_DiazCaballero_etal”].
