# Supplementary material for: Gut to lung translocation and antibiotic mediated selection shape the dynamics of Pseudomonas aeruginosa in an ICU patient
Source: Nat Commun. 2022 Nov 22;13:6523. doi: 10.1038/s41467-022-34101-2 (PMC9681761; doi:10.1038/s41467-022-34101-2)
Supplement: Supplementary file 1 — Supplementary Information [file 41467_2022_34101_MOESM1_ESM.pdf]

Supplementary Information for:

Gut to lung translocation and antibiotic mediated selection shape the dynamics of *Pseudomonas aeruginosa* in an ICU patient

Rachel M. Wheatley*[1], Julio Diaz Caballero*[1], Thomas E. van der Schalk[2,8], Fien HR De Winter[3], Liam P. Shaw[1], Natalia Kapel[1], Claudia Recanatini[4], Leen Timbermont[2], Jan Kluytmans[4], Mark Esser[5], Alicia Lacoma[6], Cristina Prat-Aymerich[4,6], Antonio Oliver[7,8], Samir Kumar-Singh[2,3], Surbhi Malhotra-Kumar[2,8], R. Craig MacLean[1]**

These authors contributed equally *
Corresponding author ** :craig.maclean@biology.ox.ac.uk

Supplementary Figure 1: Identification of genomic island.

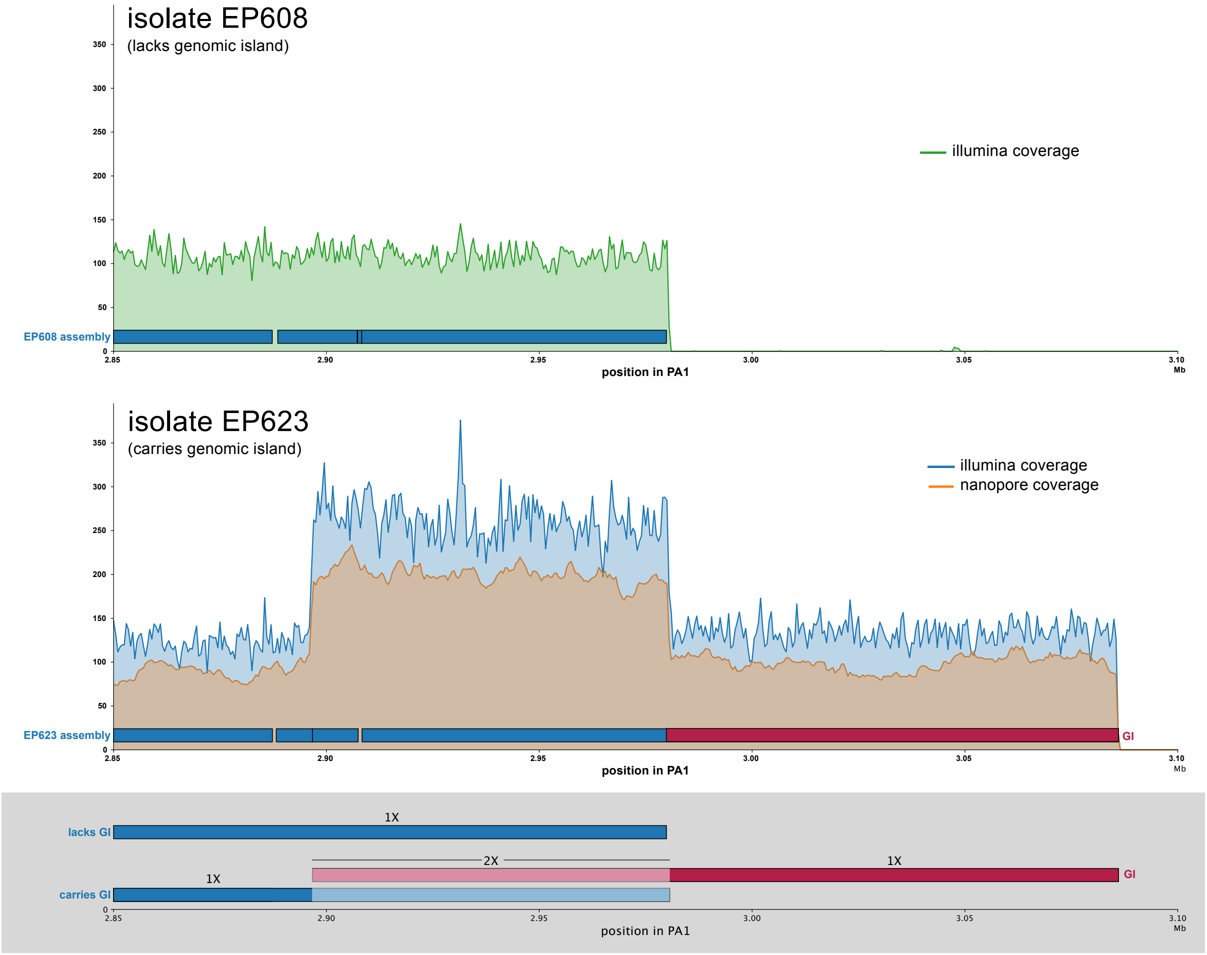

The mapping of Illumina and Oxford Nanopore sequencing data to the reference P. aeruginosa PA1 revealed isolates (e.g. EP608) that lacked a genomic region of ~125 kb. In addition, the depth of coverage of the ~75 kb upstream region was 2X greater in isolates carrying the ~125 kb region (e.g. EP623). This information revealed a ~200 kb genomic region, which carried a second copy of the ~75 kb region and additional ~125 kb genetic content.

Supplementary Figure 2: Phylogenetic tree with Bayesian dating of internal nodes.

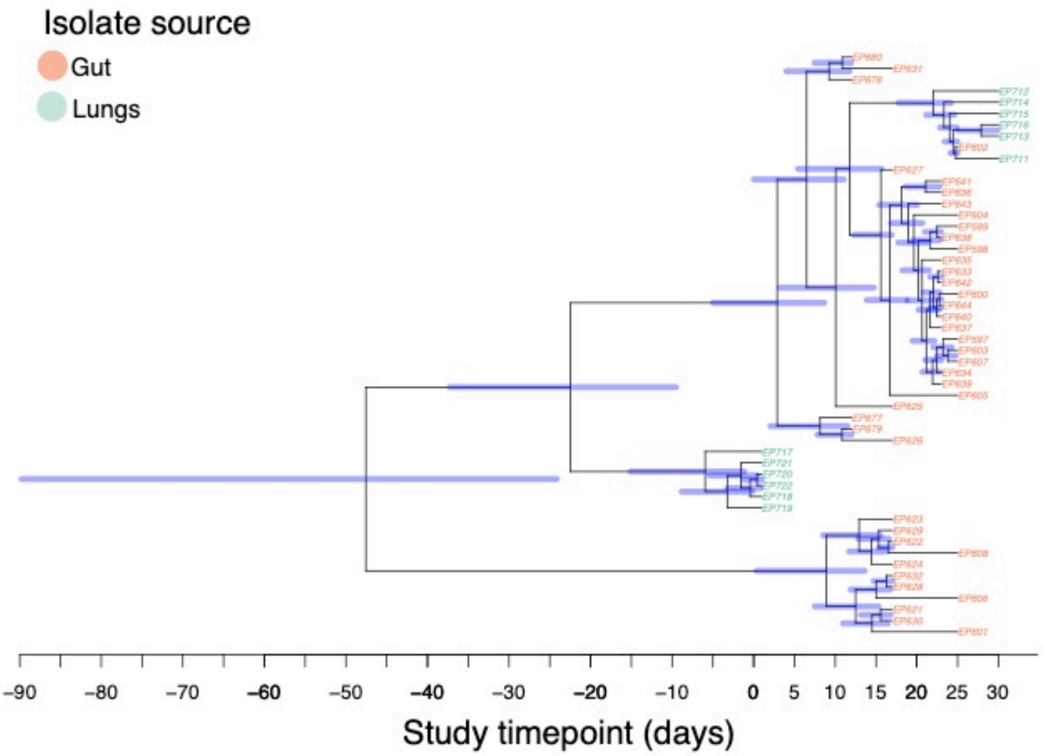

The dated genealogy (as shown in Figure 2F) showing Bayesian dating of internal nodes. Blue bars show 95% CI.

Supplementary Figure 3: Correlation between amplicon and isolate sequencing approaches.

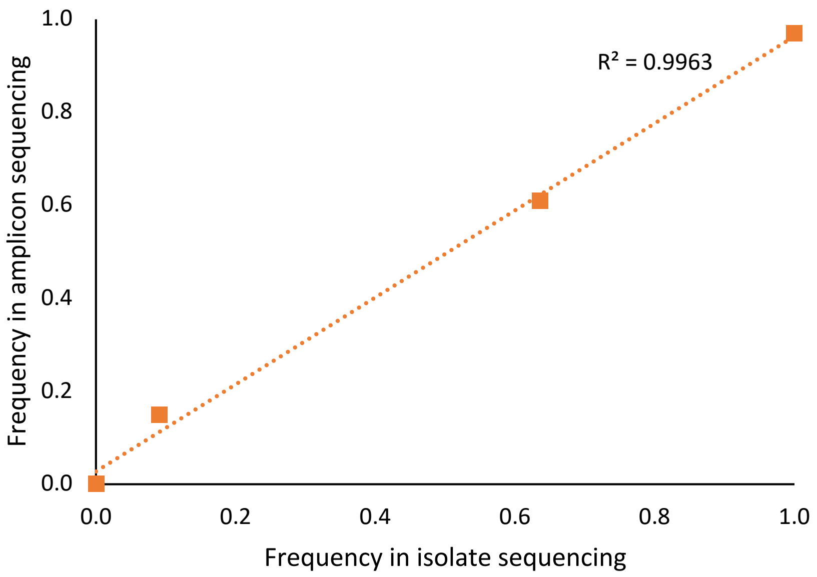

Correlation (R^2=0.9963) between the frequency of *oprD* variants measured using isolate sequencing compared to the frequency measured using amplicon sequencing. This plot shows data for the two *oprD* variants from the sampling points for which both isolate and amplicon sequencing was carried out (gut day 23 and gut day 25).

Supplementary Table 1: Genomic variants.

Variant Type	Gene Affected	Mutation	Effect
SNP	hypothetical protein	G174A	synonymous
SNP	guanine deaminase (*guaD*)	A806T	non-synonymous
SNP	methyl-accepting chemotaxis transducer (*tar*)	G392A	non-synonymous
SNP	selenocysteine-specific elongation factor (*selB*)	T1246C	non-synonymous
SNP	selenocysteine synthase (*selA*)	G447A	synonymous
SNP	intergenic		
SNP	outer membrane porin (*oprD*)	C222T	synonymous
SNP	intergenic		
SNP	anthranilate synthase component I (*trpE*)	G29A	non-synonymous
SNP	multidrug resistance operon repressor (*mexR*)	T305C	non-synonymous
SNP	hypothetical protein	G1203A	synonymous
SNP	hypothetical protein	C532T	non-synonymous
SNP	AlgW protein (*algW*)	G312A	synonymous
SNP	hypothetical protein	G354A	synonymous
SNP	LysR family transcriptional regulator (*lysR*)	C527A	non-synonymous
SNP	dehydrogenase (*betA*)	G802A	non-synonymous
SNP	two-component sensor (*pprA*)	C2217T	synonymous
Indel	intergenic		
indel	intergenic		
indel	glutamate--cysteine ligase (*gshA*)	*nt1265Δ2*	frameshift
indel	poly(beta-D-mannuronate) lyase (algL)	*nt495Δ10*	frameshift
indel	outer membrane porin (*oprD*)	*nt559Δ1*	frameshift
indel	outer membrane porin (*oprD*)	*nt1160Δ1*	frameshift
indel	DszC family monooxygenase (*dszC*)	*nt255Δ2*	frameshift

Summary of genomic variants identified within the 52 *P. aeruginosa* isolates collected over the course of the study.

Supplementary Table 2: Primer table

Primer name	Primer use	Primer Sequence	Reference
RevUL_oprD	Universal reverse primer for oprD amplicon sequencing	GTGACTGGAGTTCAGACGTGTGCTCTTCCGAT<br>CTCCATGTCACCGGTACCTACG	Adapted from Stahlberg *et al.* (2016) for use in this study.
For1L_oprD	Barcode specific forwards primer for oprD amplicon sequencing	GGACACTCTTTCCCTACACGACGCTCTTCCGAT<br>CTCACAAAGACACCATGGGAAAGAGTGTCCA<br>AGAACTAGCCGTCACTGCG	
For2L_oprD	Barcode specific forwards primer for oprD amplicon sequencing	GGACACTCTTTCCCTACACGACGCTCTTCCGAT<br>CTACAGACGACTACATGGGAAAGAGTGTCCA<br>AGAACTAGCCGTCACTGCG	
For3L_oprD	Barcode specific forwards primer for oprD amplicon sequencing	GGACACTCTTTCCCTACACGACGCTCTTCCGAT<br>CTCCTGGTAACTGGATGGGAAAGAGTGTCCA<br>AGAACTAGCCGTCACTGCG	
For4L_oprD	Barcode specific forwards primer for oprD amplicon sequencing	GGACACTCTTTCCCTACACGACGCTCTTCCGAT<br>CTTAGGGAAACACGATGGGAAAGAGTGTCCA<br>AGAACTAGCCGTCACTGCG	
For5L_oprD	Barcode specific forwards primer for oprD amplicon sequencing	GGACACTCTTTCCCTACACGACGCTCTTCCGAT<br>CTAAGGTTACACAAATGGGAAAGAGTGTCCA<br>AGAACTAGCCGTCACTGCG	
For6L_oprD	Barcode specific forwards primer for oprD amplicon sequencing	GGACACTCTTTCCCTACACGACGCTCTTCCGAT<br>CTGACTACTTTCTGATGGGAAAGAGTGTCCAA<br>GAACTAGCCGTCACTGCG	
For7L_oprD	Barcode specific forwards primer for oprD amplicon sequencing	GGACACTCTTTCCCTACACGACGCTCTTCCGAT<br>CTAAGGATTCATTCATGGGAAAGAGTGTCCAA<br>GAACTAGCCGTCACTGCG	
For8L_oprD	Barcode specific forwards primer for oprD amplicon sequencing	GGACACTCTTTCCCTACACGACGCTCTTCCGAT<br>CTACGTAACTTGGTATGGGAAAGAGTGTCCAA<br>GAACTAGCCGTCACTGCG	
For9L_oprD	Barcode specific forwards primer for oprD amplicon sequencing	GGACACTCTTTCCCTACACGACGCTCTTCCGAT<br>CTAACCAAGACTCGATGGGAAAGAGTGTCCA<br>AGAACTAGCCGTCACTGCG	
For10L_oprD	Barcode specific forwards primer for oprD amplicon sequencing	GGACACTCTTTCCCTACACGACGCTCTTCCGAT<br>CTGAGAGGACAAAGATGGGAAAGAGTGTCCA<br>AGAACTAGCCGTCACTGCG	
For11L_oprD	Barcode specific forwards primer for oprD amplicon sequencing	GGACACTCTTTCCCTACACGACGCTCTTCCGAT<br>CTTCCATTCCCTCCATGGGAAAGAGTGTCCAA<br>GAACTAGCCGTCACTGCG	
For12L_oprD	Barcode specific forwards primer for oprD amplicon sequencing	GGACACTCTTTCCCTACACGACGCTCTTCCGAT<br>CTTCCGATTCTGCTATGGGAAAGAGTGTCCAA<br>GAACTAGCCGTCACTGCG	
For13L_oprD	Barcode specific forwards primer for oprD amplicon sequencing	GGACACTCTTTCCCTACACGACGCTCTTCCGAT<br>CTAGAACGACTTCCATGGGAAAGAGTGTCCA<br>AGAACTAGCCGTCACTGCG	
For14L_oprD	Barcode specific forwards primer for oprD amplicon sequencing	GGACACTCTTTCCCTACACGACGCTCTTCCGAT<br>CTAACGAGTCTCTTATGGGAAAGAGTGTCCAA<br>GAACTAGCCGTCACTGCG	
For15L_oprD	Barcode specific forwards primer for oprD amplicon sequencing	GGACACTCTTTCCCTACACGACGCTCTTCCGAT<br>CTAGGTCTACCTCGATGGGAAAGAGTGTCCAA<br>GAACTAGCCGTCACTGCG	
For16L_oprD	Barcode specific forwards primer for oprD amplicon sequencing	GGACACTCTTTCCCTACACGACGCTCTTCCGAT<br>CTCGTCAACTGACAATGGGAAAGAGTGTCCA<br>AGAACTAGCCGTCACTGCG	

Table of primers used in this study. Adapted from primers published in: Ståhlberg, A., Krzyzanowski, P. M., Jackson, J. B., Egyud, M., Stein, L. & Godfrey, T. E. Simple, multiplexed, PCR-based barcoding of DNA enables sensitive mutation detection in liquid biopsies using sequencing. *Nucleic acids research* **44**, e105-e105 (2016).

Supplementary Table 3: Reads recovered from amplicon sequencing experiments.

Days in Study	Sample	Demultiplexed Reads	Genotyped Reads
17	lungs	427	138
20	lungs	554	208
23	gut	20,673	9,081
25	lungs	37,352	13,808
25	gut	1,699	671

Reads recovered from nanopore in amplicon sequencing experiments of *oprD.* The total demultiplexed reads and then genotyped reads for each sample are shown.
