# Supplementary material for: Gut to lung translocation and antibiotic mediated selection shape the dynamics of Pseudomonas aeruginosa in an ICU patient
Source: Nat Commun. 2022 Nov 22;13:6523. doi: 10.1038/s41467-022-34101-2 (PMC9681761; doi:10.1038/s41467-022-34101-2)
Supplement: Supplementary file 2 — Reporting Summary [file 41467_2022_34101_MOESM2_ESM.pdf]

nature portfolio

Corresponding author(s): MacLean

Last updated by author(s): Oct 9, 2022

Reporting Summary

Nature Portfolio wishes to improve the reproducibility of the work that we publish. This form provides structure for consistency and transparency in reporting. For further information on Nature Portfolio policies, see our Editorial Policies and the Editorial Policy Checklist.

Statistics

For all statistical analyses, confirm that the following items are present in the figure legend, table legend, main text, or Methods section.

n/a	Confirmed	
☐	☒	The exact sample size (*n*) for each experimental group/condition, given as a discrete number and unit of measurement
☐	☒	A statement on whether measurements were taken from distinct samples or whether the same sample was measured repeatedly
☐	☒	The statistical test(s) used AND whether they are one- or two-sided<br>*Only common tests should be described solely by name; describe more complex techniques in the Methods section.*
☒	☐	A description of all covariates tested
☒	☐	A description of any assumptions or corrections, such as tests of normality and adjustment for multiple comparisons
☐	☒	A full description of the statistical parameters including central tendency (e.g. means) or other basic estimates (e.g. regression coefficient) AND variation (e.g. standard deviation) or associated estimates of uncertainty (e.g. confidence intervals)
☐	☒	For null hypothesis testing, the test statistic (e.g. *F*, *t*, *r*) with confidence intervals, effect sizes, degrees of freedom and *P* value noted<br>*Give P values as exact values whenever suitable.*
☒	☐	For Bayesian analysis, information on the choice of priors and Markov chain Monte Carlo settings
☒	☐	For hierarchical and complex designs, identification of the appropriate level for tests and full reporting of outcomes
☒	☐	Estimates of effect sizes (e.g. Cohen's *d*, Pearson's *r*), indicating how they were calculated

Our web collection on statistics for biologists contains articles on many of the points above.

Software and code

Policy information about availability of computer code

Data collection	Data was entered in a web-based electronic data capture system that was designed for ASPIRE-ICU. The study site entered data in the system from the subject's source documents (i.e. medical chart).
Data analysis	JMP v.12 was used for statistical analysis. Trimmomatic v. 0.39, SPAdes v. 3.13.1, pilon v. 1.23, prokka v. 1.14.0, qcat v. 1.1.0, and unicycler v. 0.4.8 were used for sequencing data assembly and annotation. BWA v. 0.7.17, BCFtools v. 1.9, HaplotypeCaller of GATK v. 4.1.3.0, GenAPI v. 1.098, and breseq v. 0.34.0 were used for variant calling. BactDating v1.1.0 was used for construction of a dated isolate genealogy. Additional code used for analysis has been deposited in a community repository (GitHub: https://github.com/juliofdiaz/Wheatley_DiazCaballero_etal).

For manuscripts utilizing custom algorithms or software that are central to the research but not yet described in published literature, software must be made available to editors and reviewers. We strongly encourage code deposition in a community repository (e.g. GitHub). See the Nature Portfolio guidelines for submitting code & software for further information.

Data

Policy information about availability of data

All manuscripts must include a data availability statement. This statement should provide the following information, where applicable:

- Accession codes, unique identifiers, or web links for publicly available datasets
- A description of any restrictions on data availability
- For clinical datasets or third party data, please ensure that the statement adheres to our policy

- Source data has been deposited in a publicly available archive (Oxford Research Archive - doi: 10.5287/bodleian:r1ekRa9wE), and are available with the paper.
- Clinical data analyzed for this patient as part of the study are included in this article. Further clinical data are not publicly available for confidentiality reasons but are available upon scientific review and approval of request by the Study's Scienctific Committee.

- Isolates can be obtained from the corresponding author for research use via an MTA subject to permission from the ASPIRE research committee.
- Publicly available datasets used in study include PubMLST (last accessed on 11.06.2021)
- Code used for analysis has been deposited in a community repository (GitHub: XXXXX)
- All sequence data for the manuscript has been deposited on the NCBI short-read archive ("PRJNA802704" [https://www.ncbi.nlm.nih.gov/bioproject/PRJNA802704]).
- All sequencing data on isolates can be found within this NCBI short-read archive at:
"SRR17868883 [https://www.ncbi.nlm.nih.gov/sra/SRX14028679[accn]]"
"SRR17868884 [https://www.ncbi.nlm.nih.gov/sra/SRX14028678[accn]]"
"SRR17868885 [https://www.ncbi.nlm.nih.gov/sra/SRX14028677[accn]]"
"SRR17868886 [https://www.ncbi.nlm.nih.gov/sra/SRX14028676[accn]]"
"SRR17868887 [https://www.ncbi.nlm.nih.gov/sra/SRX14028675[accn]]"
"SRR17868888 [https://www.ncbi.nlm.nih.gov/sra/SRX14028674[accn]]"
"SRR17868889 [https://www.ncbi.nlm.nih.gov/sra/SRX14028673[accn]]"
"SRR17868890 [https://www.ncbi.nlm.nih.gov/sra/SRX14028672[accn]]"
"SRR17868891 [https://www.ncbi.nlm.nih.gov/sra/SRX14028671[accn]]"
"SRR17868892 [https://www.ncbi.nlm.nih.gov/sra/SRX14028670[accn]]"
"SRR17868893 [https://www.ncbi.nlm.nih.gov/sra/SRX14028669[accn]]"
"SRR17868894 [https://www.ncbi.nlm.nih.gov/sra/SRX14028668[accn]]"
"SRR17868895 [https://www.ncbi.nlm.nih.gov/sra/SRX14028667[accn]]"
"SRR17868896 [https://www.ncbi.nlm.nih.gov/sra/SRX14028666[accn]]"
"SRR17868897 [https://www.ncbi.nlm.nih.gov/sra/SRX14028665[accn]]"
"SRR17868898 [https://www.ncbi.nlm.nih.gov/sra/SRX14028664[accn]]"
"SRR17868899 [https://www.ncbi.nlm.nih.gov/sra/SRX14028663[accn]]"
"SRR17868900 [https://www.ncbi.nlm.nih.gov/sra/SRX14028662[accn]]"
"SRR17868901 [https://www.ncbi.nlm.nih.gov/sra/SRX14028661[accn]]"
"SRR17868902 [https://www.ncbi.nlm.nih.gov/sra/SRX14028660[accn]]"
"SRR17868903 [https://www.ncbi.nlm.nih.gov/sra/SRX14028659[accn]]"
"SRR17868904 [https://www.ncbi.nlm.nih.gov/sra/SRX14028658[accn]]"
"SRR17868905 [https://www.ncbi.nlm.nih.gov/sra/SRX14028657[accn]]"
"SRR17868906 [https://www.ncbi.nlm.nih.gov/sra/SRX14028656[accn]]"
"SRR17868907 [https://www.ncbi.nlm.nih.gov/sra/SRX14028655[accn]]"
"SRR17868908 [https://www.ncbi.nlm.nih.gov/sra/SRX14028654[accn]]"
"SRR17868909 [https://www.ncbi.nlm.nih.gov/sra/SRX14028653[accn]]"
"SRR17868910 [https://www.ncbi.nlm.nih.gov/sra/SRX14028652[accn]]"
"SRR17868911 [https://www.ncbi.nlm.nih.gov/sra/SRX14028651[accn]]"
"SRR17868912 [https://www.ncbi.nlm.nih.gov/sra/SRX14028650[accn]]"
"SRR17868913 [https://www.ncbi.nlm.nih.gov/sra/SRX14028649[accn]]"
"SRR17868914 [https://www.ncbi.nlm.nih.gov/sra/SRX14028648[accn]]"
"SRR17868915 [https://www.ncbi.nlm.nih.gov/sra/SRX14028647[accn]]"
"SRR17868916 [https://www.ncbi.nlm.nih.gov/sra/SRX14028646[accn]]"
"SRR17868917 [https://www.ncbi.nlm.nih.gov/sra/SRX14028645[accn]]"
"SRR17868918 [https://www.ncbi.nlm.nih.gov/sra/SRX14028644[accn]]"
"SRR17868919 [https://www.ncbi.nlm.nih.gov/sra/SRX14028643[accn]]"
"SRR17868920 [https://www.ncbi.nlm.nih.gov/sra/SRX14028642[accn]]"
"SRR17868921 [https://www.ncbi.nlm.nih.gov/sra/SRX14028641[accn]]"
"SRR17868922 [https://www.ncbi.nlm.nih.gov/sra/SRX14028640[accn]]"
"SRR17868923 [https://www.ncbi.nlm.nih.gov/sra/SRX14028639[accn]]"
"SRR17868924 [https://www.ncbi.nlm.nih.gov/sra/SRX14028638[accn]]"
"SRR17868925 [https://www.ncbi.nlm.nih.gov/sra/SRX14028637[accn]]"
"SRR17868926 [https://www.ncbi.nlm.nih.gov/sra/SRX14028636[accn]]"
"SRR17868927 [https://www.ncbi.nlm.nih.gov/sra/SRX14028635[accn]]"
"SRR17868928 [https://www.ncbi.nlm.nih.gov/sra/SRX14028634[accn]]"
"SRR17868929 [https://www.ncbi.nlm.nih.gov/sra/SRX14028633[accn]]"
"SRR17868930 [https://www.ncbi.nlm.nih.gov/sra/SRX14028632[accn]]"
"SRR17868931 [https://www.ncbi.nlm.nih.gov/sra/SRX14028631[accn]]"
"SRR17868932 [https://www.ncbi.nlm.nih.gov/sra/SRX14028630[accn]]"
"SRR17868933 [https://www.ncbi.nlm.nih.gov/sra/SRX14028629[accn]]"
"SRR17868934 [https://www.ncbi.nlm.nih.gov/sra/SRX14028628[accn]]"

Field-specific reporting

Please select the one below that is the best fit for your research. If you are not sure, read the appropriate sections before making your selection.

☐ Life sciences ☐ Behavioural & social sciences ☒ Ecological, evolutionary & environmental sciences

For a reference copy of the document with all sections, see nature.com/documents/nr-reporting-summary-flat.pdf

Ecological, evolutionary & environmental sciences study design

All studies must disclose on these points even when the disclosure is negative.

Study description	The study investigated the evolutionary responses to antibiotic treatment in the gut and lung samples obtained from a single patient using novel 'high definition' approaches.
Research sample	The study uses data and samples from a single patient who was was admitted to the intensive care unit (ICU) of Hospital Universitari Germans Trias i Pujol in Badalona, Spain. The patient was a 44-year-old woman admitted with seizure as the primary diagnosis. Samples of the gut and lung were collected from the patient (peri-anal swabs and endotracheal aspirate, respectively) and then processed to obtain individuals bacterial isolates (n=6-12 per sample). Choice of single patient for study relates to isolate collection (n=52) from both the gut and the lungs over longitudinal sampling in ICU (30 days), and treatment of patient with antibiotic useful for P. aeruginosa treatment (meropenem), meaning evolutionary responses to antibiotic treatment in the gut and lung samples could be investigated. We used isolates from every patient sample that was collected. In addition to isolates, DNA was extracted from some patient samples without collecting isolates. We used DNA from all patient samples where DNA was isolated.
Sampling strategy	This patient was enrolled in the ASPIRE-ICU study, and the same sampling strategy was used for all patients enrolled in the trial. Detailed information on this trial is cited in the text. Briefly, samples (endotracheal aspirate and peri-anal swabs) were collected at regularly defined intervals for all patients enrolled in the trial, and supplementary samples were collected from all patients who developed pneumonia during the trial (including this patient). Bacterial isolates collected from each clinical sample were randomly chosen isolates. As stated above, we used all available bacterial isolates and DNA samples that were collected from this patient.
Data collection	Clinical data for the patient was recorded by staff at the Germans Trias i Pujol University Hospital (Spain). Data on antibiotic use during the ICU stay and in the preceding two weeks was recorded. Respiratory samples and and peri-anal swabs were collected on the following visit days: 1 (the day of informed consent and study enrollment, 72h after ICU admission), 4, 7, and twice weekly for 30 days or until ICU discharge. For patients not diagnosed with pneumonia (this patient), bacterial isolates were only collected from the day 1 and day 30 samples.<br><br>Peri-anal swabs in skimmed milk medium and untreated respiratory samples were stored at -80 C until shipment to the Central lab at the University of Antwerp and until further analysis. Semi-quantitative culture of peri-anal swabs was performed by inoculating the swabs directly on CHROMID P. aeruginosa Agar (BioM rieux, France) and blood agar (BBL Columbia II Agar Base (BD Diagnostics, USA) supplemented with 5% defibrinated horse blood (TCS Bioscience, UK)). After incubation of 24 h at 37 C, the growth of P. aeruginosa was evaluated in four quadrants. Plates without growth were further incubated for 48 h and 72 h.<br><br>Patient endotracheal aspirate samples were blended (30,000 rpm, probe size 8 mm, steps of 10 s, max 60 s in total), diluted 1:1 v/v with Lysomucil (10% Acetylcysteine solution) (Zambon S.A, Belgium) and incubated for 30 minutes at 37 C with 10 s vortexing every 15 minutes. Thereafter, quantitative culture was performed by inoculating 10-fold dilutions on CHROMID P. aeruginosa Agar and blood agar using spiral plater EddyJet (IUL, Spain). Plates were incubated at 37 C for 24 h and CFU/mL was calculated. Plates without growth were further incubated for 48 h and 72 h. Matrix-Assisted Laser Desorption Ionization-Time of Flight Mass Spectrometry (MALDI-TOF MS) was used to identify Pseudomonas aeruginosa isolates which were stored at -80 C until further use.
Timing and spatial scale	Peri-anal samples were collected at days 1, 4, 7, 9, 12, 17, 19, 23, 25 and 30. Endotracheal aspirate samples were collected at days 1, 4, 7, 9, 12, 17, 19, 24 and 30. Actual dates will not be provided as these could compromise patient anonymity in conjunction with data reported in the manuscript. A single peri-anal swab and ETA sample were taken at each time point, and we have assumed that these are representative of the gut and lung populaitons, respectively.
Data exclusions	No data was excluded from the analysis.
Reproducibility	All experiments reported in this manuscript were repeated. Antibiotic susceptibility tests were carried out in 3 independent assays, aerobic growth assays were carried out in a minimum of 7 independent assays, anaerobic growth assays were carried out in a minimum of 3 independent assays. All attempts at replication were successful.
Randomization	Aerobic growth assays: we measured the growth of all isolates in replicate assays.<br>Anaerobic growth assays: we measured the growth of a minimum of three randomly selected isolates from each phylogeny group, with three replicate assays. The isolates from each phylogeny group were selected by random number generation in excel (=RAND()).<br>MIC assays: we assayed the antibiotic susceptibility to meropenem of all isolates in three replicate assays.
Blinding	All bacterial isolates were assigned a unique identifier that did not reflect the sample type (ie endotracheal aspirate or peri-anal), or time point.

Did the study involve field work? ☐ Yes ☒ No

Reporting for specific materials, systems and methods

We require information from authors about some types of materials, experimental systems and methods used in many studies. Here, indicate whether each material, system or method listed is relevant to your study. If you are not sure if a list item applies to your research, read the appropriate section before selecting a response.

Materials & experimental systems

n/a	Involved in the study
☒	☐ Antibodies
☒	☐ Eukaryotic cell lines
☒	☐ Palaeontology and archaeology
☒	☐ Animals and other organisms
☐	☒ Human research participants
☐	☒ Clinical data
☒	☐ Dual use research of concern

Methods

n/a	Involved in the study
☒	☐ ChIP-seq
☒	☐ Flow cytometry
☒	☐ MRI-based neuroimaging

Human research participants

Policy information about studies involving human research participants

Population characteristics

This study focuses on one single patient, who was enrolled in the study as part of a cohort of adult patients admitted to the ICU with an anticipated length of stay of 48 hours or longer who were undergoing mechanical ventilation at ICU admission. S. aureus colonization was ascertained in the nose and lower respiratory tractof all eligible participants; S. aureus–colonized and non-colonized patients were then enrolled into the study cohort in a 1:1 ratio. The patient was a 44-year-old woman admitted with seizure as the primary diagnosis.

Recruitment

All consecutive adult ICU patients fulfilling inclusion criteria were approached for participation in the ASPIRE-ICU study (full details of ASPIRE-ICU study are publicly available here: https://www.ncbi.nlm.nih.gov/pmc/articles/PMC5613521/).
The study is composed of two study populations, the surveillance population and study cohort population. The study cohort is nested within the larger surveillance population; this means that all data and specimens collected specifically for study cohort participants is in addition to data already captured by ways of surveillance. Patients eligible to participate in the surveillance population must be on mechanical ventilation (MV) upon or (expected to be) within 24 h after ICU admission and have an expected length of stay (LOS) of at least 48 h. Patients with an expected ICU stay of less than 48 h are at a lower risk for developing ICU infections since this population is generally healthier, without significant comorbidities and shorter in the ICU. Surveillance patients that meet the eligibility criteria described below for the study cohort population will be enrolled. 2000 study cohort subjects are required to meet the objectives of this study.
Inclusion criteria for study cohort
-Participant is 18 years or older at the time of enrollment.
-Participant is on mechanical ventilation at ICU admission, or is (expected to be) within 24 h thereafter, based on investigator's judgment.
-Expected stay in ICU is 48 h or longer based on investigator's judgment.
-S. aureus colonization status is known within 72 h after start of first episode of mechanical ventilation and according to the result, the patient qualifies for enrollment.
-Written informed consent from subject / legally accepted representative within 72 h after start of first episode of mechanical ventilation.

Exclusion criteria for study cohort
-Previous participation as a subject in the study cohort of this study.
-Simultaneous participation of the subject in any preventive experimental study into anti-staphylococcus or anti-pseudomonas aeruginosa interventions.
-Expected death (moribund status) within 48 h, or ICU discharge of the participant within 24 h, at the moment of informed consent.

The subject described in the this study was one of the study cohort participants recruited at the Germans Trias i Pujol University Hospital (Badalona, Spain).

Ethics oversight

The study protocol was approved by the Research Ethics Committee of the Germans Trias i Pujol University Hospital.

Note that full information on the approval of the study protocol must also be provided in the manuscript.

Clinical data

Policy information about clinical studies
All manuscripts should comply with the ICMJE guidelines for publication of clinical research and a completed CONSORT checklist must be included with all submissions.

Clinical trial registration

ASPIRE-ICU ClinicalTrials.gov Identifier: NCT02413242

Study protocol

Paling, F.P., et al., Rationale and design of ASPIRE-ICU: a prospective cohort study on the incidence and predictors of Staphylococcus aureus and Pseudomonas aeruginosa pneumonia in the ICU. BMC infectious diseases, 2017. 17(1): p. 643 (the full ASPIRE-ICU protocol can be obtained upon request).

Data collection

ASPIRE-ICU was a cohort study of adult ICU patients at 30 hospitals in 11 European countries that recruited participants between June 2015 and October 2018. The subject described in the this study was one of the participants recruited at the Germans Trias i Pujol University Hospital (Badalona, Spain). Actual dates of ICU admission for this patient will not be provided as these could compromise patient anonymity in conjunction with data reported in the manuscript.

Outcomes

The primary outcome (Pseudomonas aeruginosa pneumonia acquired in the ICU) was assessed in multiple steps. First, the following 4 clinical criteria were assessed daily: any new antibiotic use, new blood cultures performed, new chest radiograph or computed tomography scan that shows a new or worsening infiltrate, or other new reason to suspect pneumonia. In cases of at positive answer, a combination of objective major and minor criteria was assessed to categorize patients as having protocol-defined pneumonia or not, as described elsewhere. The primary end point was determined post hoe on the basis of isolation of P. aeruginosa from any lower respiratory tract specimen (including both clinical and study surveillance cultures) or blood culture in the 3 days before and after the day of pneumonia diagnosis, according to clinical criteria. Death was assessed at ICU discharge, at day 30 and day 90 after ICU admission. No other secondary outcomes were evaluated. From this evaluation, the patient in this study were not determined to have Pseudomonas aeruginosa pneumonia acquired in the ICU.
